# Pericytes Favor Oligodendrocyte Fate Choice in Adult Neural Stem Cells

**DOI:** 10.3389/fncel.2019.00085

**Published:** 2019-03-27

**Authors:** Maria Elena Silva, Simona Lange, Bryan Hinrichsen, Amber R. Philp, Carolina R. Reyes, Diego Halabi, Josselyne B. Mansilla, Peter Rotheneichner, Alerie Guzman de la Fuente, Sebastien Couillard-Despres, Luis F. Bátiz, Robin J. M. Franklin, Ludwig Aigner, Francisco J. Rivera

**Affiliations:** ^1^Laboratory of Stem Cells and Neuroregeneration, Institute of Anatomy, Histology and Pathology, Faculty of Medicine, Universidad Austral de Chile, Valdivia, Chile; ^2^Center for Interdisciplinary Studies on the Nervous System (CISNe), Universidad Austral de Chile, Valdivia, Chile; ^3^Institute of Pharmacy, Faculty of Sciences, Universidad Austral de Chile, Valdivia, Chile; ^4^Institute of Molecular Regenerative Medicine, Paracelsus Medical University, Salzburg, Austria; ^5^Spinal Cord Injury and Tissue Regeneration Center Salzburg (SCI-TReCS), Paracelsus Medical University, Salzburg, Austria; ^6^Institute of Experimental Neuroregeneration, Paracelsus Medical University Salzburg, Salzburg, Austria; ^7^Wellcome Trust and Medical Research Council (MRC) Cambridge Stem Cell Institute & Department of Clinical Neurosciences, University of Cambridge, Cambridge, United Kingdom; ^8^Austrian Cluster for Tissue Regeneration, Vienna, Austria; ^9^Centro de Investigación Biomédica (CIB), Facultad de Medicina, Universidad de los Andes, Santiago, Chile

**Keywords:** pericytes, neural stem cells, oligodendrogenesis, Lama2, remyelination, vascular niche

## Abstract

Multiple sclerosis (MS) is an inflammatory demyelinating disease of the central nervous system (CNS). Upon demyelination, oligodendrocyte progenitor cells (OPCs) are activated and they proliferate, migrate and differentiate into myelin-producing oligodendrocytes. Besides OPCs, neural stem cells (NSCs) may respond to demyelination and generate oligodendrocytes. We have recently shown that CNS-resident pericytes (PCs) respond to demyelination, proliferate and secrete Laminin alpha2 (Lama2) that, in turn, enhances OPC differentiation. Here, we aimed to evaluate whether PCs influence the fate choice of NSCs *in vitro*, towards the production of new myelin-producing cells. Indeed, upon exposure to conditioned medium derived from PCs (PC-CM), the majority of NSCs gave rise to GalC- and myelin basic protein (MBP)-expressing oligodendrocytes at the expense of the generation of GFAP-positive astrocytes. Consistent with these findings, PC-CM induces an increase in the expression of the oligodendrocyte fate determinant Olig2, while the expression level of the astrocyte determinant ID2 is decreased. Finally, pre-incubation of PC-CM with an anti-Lama2 antibody prevented the generation of oligodendrocytes. Our findings indicate that PCs-derived Lama2 instructs NSCs to an oligodendrocyte fate choice favoring the generation of myelin-producing cells at the expense of astrocytes *in vitro*. Further studies aiming to reveal the role of PCs during remyelination may pave the way for the development of new therapies for the treatment of MS.

## Introduction

In multiple sclerosis (MS) myelin loss within the central nervous system (CNS) represents a crucial pathological event that, when persistent, results in irreversible axonal death leading to a decline in neurological function. Myelin sheaths are restored through a process known as remyelination, which is feasible due to the presence of CNS-resident oligodendrocyte progenitor cells (OPCs). Upon demyelination, OPCs become activated and they proliferate, migrate, and differentiate into remyelinating oligodendrocytes (Franklin and ffrench-Constant, 2008; Zawadzka et al., 2010). Although this spontaneous reparative phenomenon is quite robust, it often fails in the more advanced stages of MS (Blakemore, 1974; Wolswijk, 1998). Thus, revealing the mechanisms that underlie OPC-mediated remyelination or exploring alternative remyelination sources in the CNS might provide insights for the development of regenerative MS therapies.

Several studies have identified positive and negative regulators of myelin repair such as, secreted growth factors, members of the extracellular matrix (ECM) as well as cells from the non-oligodendroglial lineage (Rivera et al., 2010). There is increasing evidence that pericytes (PCs) might have essential functions in CNS repair and, particularly during remyelination. PCs are perivascular cells located at the abluminal surface of capillaries that regulate angiogenesis, endothelial cell function and control microvascular blood flow (Armulik et al., 2010). Within the CNS, pericytes are essential for the proper stability and function of the blood-brain-barrier (BBB; Winkler et al., 2011). We have recently demonstrated that after demyelination, PCs promote oligodendroglial differentiation of OPCs through the expression/secretion of Laminin alpha 2 (Lama2; De La Fuente et al., 2017). We now aim to explore if PCs might also influence other CNS-resident progenitor cells.

OPCs are not the only source of oligodendrocytes. Adult neural stem cells (NSCs) located in specific neurogenic niches respond to demyelination and contribute to the generation of new oligodendrocytes. In response to myelin loss, CNS-resident NSCs proliferate and give rise to Olig2-expressing cells that migrate towards the lesion site and differentiate into oligodendrocytes (Nait-Oumesmar et al., 1999; Menn et al., 2006; Etxeberria et al., 2010; Jablonska et al., 2010; Kazanis et al., 2017). Although there is compelling evidence showing that adult NSCs may change their neurogenic fate towards the generation of oligodendrocytes, the molecular and cellular mechanisms that enable this phenomenon, however, remain elusive. Here, we examined the possibility of soluble factors derived from PCs (particularly, Lama2) to induce adult NSCs to generate oligodendrocytes.

## Materials and Methods

### Preparation of Primary CNS Pericytes

**Figure 1:**
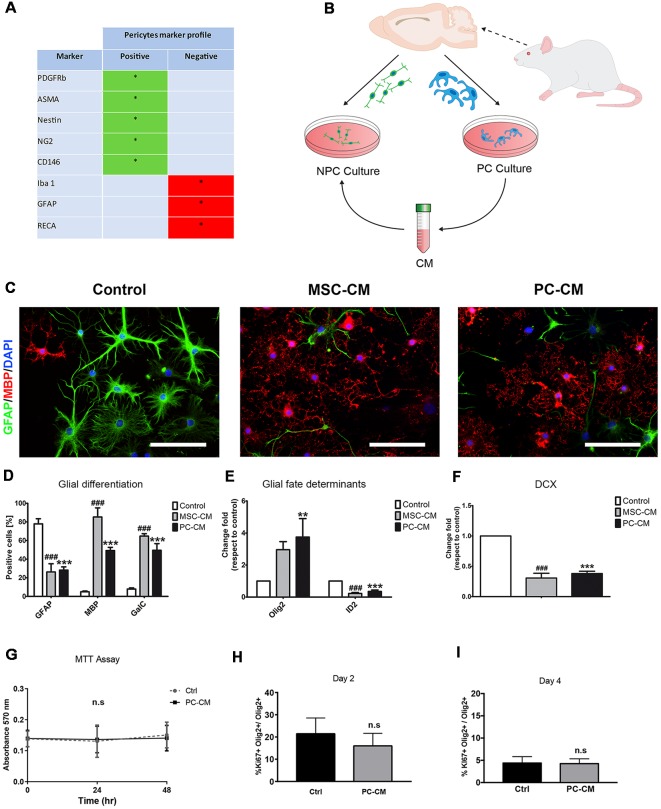
Pericytes (PCs) promote oligodendrocyte differentiation in adult neural stem cells (NSCs). **(A)** Table shows the qualitative profile expression of cell markers in PC cultures** (B)** Schematic representation of the experimental design. **(C)** Fluorescence images displaying GFAP+/myelin basic protein (MBP+) cells after 7 days and **(D)** their respective graph. Scale bars in **(B)** = 100 μm. Note the increase in the percentage of MBP-expressing oligodendrocytes and the reduction in the proportion of GFAP+ astrocytes when NSCs were exposed to PC-CM. Quantitative PCR analysis of the expression of **(E)** glial fate determinants (Olig2 for oligodendrocytes and ID2 for astrocytes) and of **(F)** the neurogenic marker doublecortin (DCX). Note that PC-CM increases the expression of Olig2 and decreases ID2 while DCX was not affected. Means ± SD are shown. Data were obtained from three independent experiments and were analyzed by analysis of variance (ANOVA) followed by Tukey *post hoc* test. **p < 0.01, ^###^ and ***p < 0.001. *Indicates statistical difference for PC-CM and ^#^ for MSC-CM. **(G)** Graph of MTT proliferation assay after incubation with alfa MEM (Ctrl) and PC-CM during 0, 24 and 48 h showing that there are no significant differences between the two conditions. **(H,I)** Quantitative analysis of Ki-67 + Olig2+ cells compared to the total population of Olig2 + cells 2 and 4 days after incubation with alfa MEM (Ctrl) and PC-CM. Data show that there are no significant differences between the two conditions and the two time points considered; ns, not significant.

Preparation was performed as in De La Fuente et al. (2017). Briefly, rat primary PCs were isolated from 6–8 weeks old female Fisher 344 rats. Brains were collected in ice cold alphaMEM (Gibco). Meninges were removed and brains were minced in alphaMEM and overlayed on a cold 15% dextran solution and centrifuged at 5,000 *g* at 4°C for 10 min. Pellet containing cerebral vessels was collected, washed, resuspended in alphaMEM, and then filtered through a 150 μm mesh. The flow-through was again filtered through a 40 μm mesh to eliminate single cells. Microvessels were then collected and digested in a waterbath for 30 min at 37°C. Digested microvessels were centrifuged (1,000 *g*, 10 min), cells were cultured in alphaMEM containing 20% FBS (Gibco) and 1% Penicillin/ Streptomycin (Thermo Fisher, Waltham, MA, USA) in a humidifying incubator (20% O_2_, 5% CO_2_ at 37°C). After first passage cells were incubated in alphaMEM containing 10% FBS. Expanded cells displayed a typical PC marker expression profile, confirming the identity and purity of this culture (Figure 1A).

### Preparation of Primary Neural Stem Cells

Rat-derived hippocampal NSC preparation was performed as previously described (Wachs et al., 2003). Briefly, 6–8 weeks old female Fisher 344 rats were anesthetized with Isofluran and subsequently decapitated. Hippocampus were dissected and collected in ice cold DPBS/glu. Brain regions were minced and enzymatically digested, washed, and cultured in NBA medium (Gibco) containing EGF and FGF for sphere formation. Five rats were used for one preparation. After preparation, NSCs were cultured in T25 flasks in a humidifying incubator (20% O_2_, 5% CO_2_ at 37°C).

### NSC Stimulation With PC-CM and MSC-CM

NSCs were treated with PC-CM and MSC-CM as previously described (Rivera et al., 2006). Briefly, NSCs were seeded overnight onto poly-ornithine (250 μg/mL) and laminin (5 μg/mL)-coated glass coverslips at a density of 12,000 cells/cm^2^ in αMEM-10% FBS. Next, media was replaced, and cells were incubated either with PC-CM or MSC-CM. NSCs were alternatively incubated in αMEM-10% FBS as a control condition. Media was changed every third day. After 1 week of incubation, cells were fixed for 10 min with phosphate-buffered 4% paraformaldehyde (Sigma-Aldrich, Taufkirchen, Germany) and processed for immunofluorescence.

### Blocking of Lama2 in PC-CM

PC-CM was collected as previously described (De La Fuente et al., 2017). To block Lama2 in the PC-CM, the media was incubated in rotation for 2 h at room temperature and covered from light with 1:50 dilution of either general rabbit IgG antibody (Cell Signaling, 2729S) or rabbit anti-Lama2 antibody (Santa Cruz, SC-20412). Upon incubation, the NSC medium was replaced by the PC-CM (pre-incubated with the different antibodies). This process was repeated after 3 days of stimulation and the experiment was stopped at 7 days of incubation.

### Immunocytochemistry

Immunocytochemical stainings were performed as previously described (Steffenhagen et al., 2012). The following antibodies and dilutions were used. Primary antibodies: rabbit anti-GFAP 1:1,000 (Dako, Denmark); mouse anti-myelin basic protein (anti-MBP) 1:750 (SMI-94, Covance, Anopoli Biomedical Systems, Eichgraben, Austria); rabbit anti-Galactocerebroside (GalC) 1:200 (Millipore, Burlington, MA, USA); rabbit anti Ki67 1:500 (Thermos Sc.); goat anti olig2 1:200 (Abcam); Secondary antibodies: donkey anti-mouse, rabbit, goat conjugated with Alexa Fluor 488, Alexa 568 1:1,000 (Molecular Probes, Eugene, OR, USA). Nuclear counterstaining was performed with 4′,6′-diamidino-2-phenylindole dihydrochloride hydrate at 0.25 μg/μl (DAPI; Sigma, Germany). For more details see Supplementary Materials.

### Statistical Analysis

**Figure 2:**
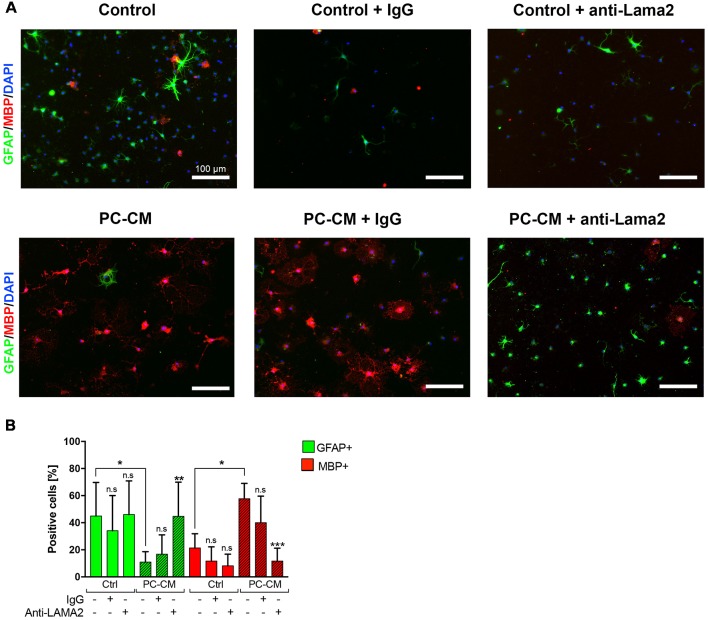
PC-derived Laminin alpha2 (Lama2) induces oligodendrocyte fate decision NSCs. **(A)** NSCs were cultured for seven days with control media or PC-CM that, alternatively, were pre-treated with the addition of general Rabbit-IgG or anti-Lama2 anti-bodies. **(B)** Percentage of GFAP-expressing astrocytes (*n* = 5) and MBP+ oligodendrocytes (*n* = 5) according to the treatment depicted in **(A)**. Scale bars, 100 μm. Note that the anti-Lama2 antibody blocks the oligodendrogenic effect and suppresses the astrogenic inhibition induced by PC-CM in NSCs. Means ± SD are shown. Data were obtained from two independent experiments and were analyzed by two-way ANOVA (uncorrected Fisher’s LSD). *p < 0.05, ***p < 0.001 compared to the respective untreated media control; ns, not significant.

Data are presented as means ± SD and statistical analysis was performed using PRISM4 (GraphPad, San Diego, CA, USA). *P* values of < 0.05 were considered significant. Data from Figures 1D–G were analyzed using a one-way analysis of variance (ANOVA) and Tukey *post hoc* considering three biologically independent experiments. Data from Figures 1H,I were analyzed using an unpaired *t*-test. Data from Figure 2 were analyzed using a two-way ANOVA (uncorrected Fisher’s LSD) considering five biologically independent experiments which were performed in technical triplicate. Data from Figures 1H,I were analyzed using an unpaired *t-test*. Each biological experiment was performed in technical triplicates.

## Results

### Soluble Factors Released by PCs Induce Oligodendrocyte Fate Decision on Adult NSCs

Since mesenchymal stem cells (MSCs) secrete soluble factors that instruct oligodendrocyte fate choice in adult NSCs (Rivera et al., 2006) and PCs display similar cellular features to MSCs (Crisan et al., 2008), we evaluated whether conditioned medium derived from PCs (PC-CM) also influences NSC function. Conditioned medium derived from MSCs (MSC-CM) was used as positive control (Rivera et al., 2006; De La Fuente et al., 2017). PC-CM significantly induced the expression of oligodendroglial markers in adult NSCs cultures. After 7 days of exposure to PC-CM, 49 ± 6% of the NSCs were positive for MBP while only 5 ± 2% were positive under control conditions (Figures 1B–D). Similarly, PC-CM elevated the percentage of GalC positive cells in NSCs. There was no statistical difference between the proportion of GalC+ and MBP-expressing cells, suggesting that PC-CM promotes the generation of fully differentiated oligodendrocytes. Nevertheless, it seems that MSC-CM displays a stronger oligodendrogenic effect on NSCs since more MBP-expressing cells are generated compared to PC-CM. This increase in oligodendrogenesis induced by PC-CM was accompanied by a significant decrease in the generation of GFAP-expressing astrocytes compared to control conditions. mRNA expression analyses of the glial fate determinants Olig2 and Id2 (Samanta and Kessler, 2004; Steffenhagen et al., 2012) by qRT-PCR supported these findings. Upon 3 days of exposure to PC-CM, there was a significant increase in Olig2 mRNA expression (3.8 ± 1.2-fold) as well as a reduction in mRNA expression levels of the astrocyte determinant Id2 (0.4 ± 0.1-fold) compared to control conditions (Figure 1D). PC-CM also reduced neuronal fate in NSCs, since doublecortin (DCX) mRNA expression was significantly decreased (0.4 ± 0.1) relative to control conditions (Figure 1E). These data indicate that soluble factors derived from PCs induce an oligodendrocyte fate in adult NSCs at the expense of astroglial and neuronal fate. However, this increase in the generation of oligodendrocytes induced by PC-CM may be due to an indirect effect on cell viability/proliferation. To exclude this possibility, we performed an MTT assay and found no differences between PC-CM and control conditions (Figure 1G). In addition, to discard that PC-CM may specifically increase the proliferation of NSC-derived oligodendrocytes we evaluated the proportion of Ki67+ cells among the Olig2-expressing population and found no differences between the tested conditions (Figures 1H,I). Together, these data indicate that soluble factors derived from PCs do not affect cell viability/proliferation but directly favor oligodendrocyte fate choice and differentiation of NSCs.

### PCs-Derived Lama2 Favors Oligodendrocyte Differentiation at Expense of Astrogenesis in NSCs

Since we have previously shown that Lama2 secreted by PCs enhances OPC differentiation during remyelination (De La Fuente et al., 2017), we tested whether Lama2 is also responsible for the oligodendrocyte fate choice induced by PCs in NSCs. As observed in Figure 1, treatment of NSCs with PC-CM induced oligodendrocyte fate choice, however, pre-treatment of PC-CM with an anti-Lama2 blocking antibody led to a significant reduction in MBP-expressing oligodendrocytes compared to the PC-CM media control (11.7 ± 9.5% vs. 56.9 ± 12.9% respectively) and a significant increase in the percentage of GFAP+ astrocytes (44, 7 ± 25.0% vs. 7.5 ± 3.6%, respectively; Figures 2A,B). In fact, there was no significant difference in the amount of GFAP+ and MBP+ cells between control media and PC-CM + anti-Lama2 conditions. Thus, pre-incubation of PC-CM with an anti-Lama2 antibody prevents the generation of oligodendrocytes indicating that Lama2 secreted by PCs induces the fate and differentiation of NSCs towards oligodendrocytes.

## Discussion

Besides neurovascular homeostasis, PCs are involved in CNS repair and regeneration (Lange et al., 2013), particularly, contributing to oligodendrocyte differentiation during remyelination (De La Fuente et al., 2017). The latter involves remodeling of ECM composition, i.e., secretion of Lama2. Here, we demonstrate that this PC activity is not restricted to OPCs but also influences NSC fate choice and differentiation. This might be relevant in CNS remyelination. For example, upon demyelination in the corpus callosum (CC), NSCs located at the subependymal zone (SEZ) of the lateral ventricles proliferate and migrate towards the lesion site and differentiate into oligodendrocytes (Menn et al., 2006; Etxeberria et al., 2010; Jablonska et al., 2010; Kazanis et al., 2017). Therefore, PCs and PC-derived Lama2 may drive this oligodendrocyte fate decision in SEZ-resident NSCs contributing to newly generated oligodendrocytes in the CC, however, this assumption should be further studied in an *in vivo* model.

A number of studies have recently addressed the impact of PCs in CNS pathologies (reviewed in Cheng et al., 2018). PC deficiency impairs the generation of new oligodendrocytes during CNS remyelination (De La Fuente et al., 2017) and human demyelinating MS lesions show a reduction in pericytes number (Iacobaeus et al., 2017). These observations together with our recent findings strongly suggest a regenerative role of these perivascular cells during MS progression and, therefore, making PCs and Lama2 interesting therapeutic targets for the development of future MS therapies (Azevedo et al., 2018).

In summary, we demonstrated that Lama2 secreted by PCs induces oligodendrocyte fate choice and differentiation of adult NSCs while it decreases the generation of astrocytes. Further studies are mandatory to determine the role of PCs during CNS remyelination in more detail and to pave the way towards the development of pro-remyelinating therapies for the treatment of MS.

## Ethics Statement

All experiments were conducted in accordance with the Chilean Government’s Manual of Bioethics and Biosafety (CONICYT: the Chilean Commission of Scientific and Technological Research, Santiago of Chile, Chile) and according to the guidelines established by the Animal Protection Committee of the Universidad Austral de Chile (Approved Number: informe 258/2016). Animals were handled in accordance with the guidelines of the National Institutes of Health Guide for Care and Use of Laboratory Animals and approved by the Institutional Animal Care and Use Ethics Committee of the Universidad Austral de Chile. In addition, animal handling for primary cell culture preparations was also performed in accordance with Austrian laws on animal experimentation and were approved by Austrian regulatory authorities (Permit No. BMWF-66.012/0001-II/3b/2014; license codes BMBF-66-012/0037-WF/V/3b/2014 and BMWF-66.012/0032-WF/V/3b/2015).

## Author Contributions

MS, SL and FR conceived the project. MS, BH, SL, AP, AGF, RF, LA and FR designed the study. MS and FR wrote the manuscript. SL, DH, RF, AGF and LA edited the manuscript. MS, BH, SL, DH and FR designed the figures. SL, MS, BH, AP, AGF and FR planned the experiments. MS, BH, SL, AP, CR, JM and PR, conducted the experiments. MS, BH, SL, AP, CR, JM and PR collected data. MS, BH, SL, AP, CR, JM, DH and FR analyzed data. MS, BH, SL, AGF, SC-D, LB, RF, LA and FR interpreted data. FR supervised the project. LA, RF and FR supported this study financially. All authors read and commented on the manuscript.

## Conflict of Interest Statement

The authors declare that the research was conducted in the absence of any commercial or financial relationships that could be construed as a potential conflict of interest.

## References

[B1] Armulik A. Genové G. Mäe M. Nisancioglu M. H. Wallgard E. Niaudet C.. (2010). Pericytes regulate the blood-brain barrier. Nature 468, 557–561. 10.1038/nature09522 20944627

[B2] Azevedo P. O. Sena I. F. G. Andreotti J. P. Carvalho-Tavares J. Alves-Filho J. C. Cunha T. M.. (2018). Pericytes modulate myelination in the central nervous system. J. Cell. Physiol. 233, 5523–5529. 10.1002/jcp.26348 29215724 PMC6076852

[B3] Blakemore W. F. (1974). Pattern of remyelination in the CNS. Nature 249, 577–578. 10.1038/249577a0 4834082

[B4] Cheng J. Korte N. Nortley R. Sethi H. Tang Y. Attwell D. (2018). Targeting pericytes for therapeutic approaches to neurological disorders. Acta Neuropathol. 136, 507–523. 10.1007/s00401-018-1893-0 30097696 PMC6132947

[B5] Crisan M. Yap S. Casteilla L. Chen C.-W. Corselli M. Park T. S.. (2008). A perivascular origin for mesenchymal stem cells in multiple human organs. Cell Stem Cell 3, 301–313. 10.1016/j.stem.2008.07.003 18786417

[B6] De La Fuente A. G. Lange S. Silva M. E. Gonzalez G. A. Tempfer H. van Wijngaarden P.. (2017). Pericytes stimulate oligodendrocyte progenitor cell differentiation during CNS remyelination. Cell Rep. 20, 1755–1764. 10.1016/j.celrep.2017.08.007 28834740 PMC5574064

[B7] Etxeberria A. Mangin J. M. Aguirre A. Gallo V. (2010). Adult-born SVZ progenitors receive transient synapses during remyelination in corpus callosum. Nat. Neurosci. 13, 287–289. 10.1038/nn.2500 20173746 PMC4681435

[B8] Franklin R. J. M. ffrench-Constant C. (2008). Remyelination in the CNS: from biology to therapy. Nat. Rev. Neurosci. 9, 839–855. 10.1038/nrn2480 18931697

[B9] Iacobaeus E. Sugars R. V. Törnqvist Andrén A. Alm J. J. Qian H. Frantzen J.. (2017). Dynamic changes in brain mesenchymal perivascular cells associate with multiple sclerosis disease duration, active inflammation, and demyelination. Stem Cells Transl. Med. 6, 1840–1851. 10.1002/sctm.17-0028 28941240 PMC6430046

[B10] Jablonska B. Aguirre A. Raymond M. Szabo G. Kitabatake Y. Sailor K. A.. (2010). Chordin-induced lineage plasticity of adult SVZ neuroblasts after demyelination. Nat. Neurosci. 13, 541–550. 10.1038/nn.2536 20418875 PMC4059417

[B11] Kazanis I. Evans K. A. Andreopoulou E. Dimitriou C. Koutsakis C. Karadottir R. T.. (2017). Subependymal zone-derived oligodendroblasts respond to focal demyelination but fail to generate myelin in young and aged mice. Stem Cell Reports 8, 685–700. 10.1016/j.stemcr.2017.01.007 28196689 PMC5355571

[B12] Lange S. Trost A. Tempfer H. Bauer H. C. Bauer H. Rohde E.. (2013). Brain pericyte plasticity as a potential drug target in CNS repair. Drug Discov Today 18, 456–463. 10.1016/j.drudis.2012.12.007 23266366

[B13] Menn B. Garcia-Verdugo J. M. Yaschine C. Gonzalez-Perez O. Rowitch D. Alvarez-Buylla A. (2006). Origin of oligodendrocytes in the subventricular zone of the adult brain. J. Neurosci. 26, 7907–7918. 10.1523/JNEUROSCI.1299-06.2006 16870736 PMC6674207

[B14] Nait-Oumesmar B. Decker L. Lachapelle F. Avellana-Adalid V. Bachelin C. Van Evercooren A. B. (1999). Progenitor cells of the adult mouse subventricular zone proliferate, migrate and differentiate into oligodendrocytes after demyelination. Eur. J. Neurosci. 11, 4357–4366. 10.1046/j.1460-9568.1999.00873.x 10594662

[B15] Rivera F. J. Couillard-Despres S. Pedre X. Ploetz S. Caioni M. Lois C.. (2006). Mesenchymal stem cells instruct oligodendrogenic fate decision on adult neural stem cells. Stem Cells 24, 2209–2219. 10.1634/stemcells.2005-0614 16763198

[B16] Rivera F. J. Steffenhagen C. Kremer D. Kandasamy M. Sandner B. Couillard-Despres S.. (2010). Deciphering the oligodendrogenic program of neural progenitors: cell intrinsic and extrinsic regulators. Stem Cells Dev. 19, 595–606. 10.1089/scd.2009.0293 19938982

[B17] Samanta J. Kessler J. A. (2004). Interactions between ID and OLIG proteins mediate the inhibitory effects of BMP4 on oligodendroglial differentiation. Development 131, 4131–4142. 10.1242/dev.01273 15280210

[B18] Steffenhagen C. Dechant F. X. Oberbauer E. Furtner T. Weidner N. Küery P.. (2012). Mesenchymal stem cells prime proliferating adult neural progenitors towards an oligodendrocyte fate. Stem Cells Dev. 21, 1838–1851. 10.1089/scd.2011.0137 22074360 PMC3396148

[B19] Wachs F. P. Couillard-Despres S. Engelhardt M. Wilhelm D. Ploetz S. Vroemen M.. (2003). High efficacy of clonal growth and expansion of adult neural stem cells. Lab. Invest. 83, 949–962. 10.1097/01.lab.0000075556.74231.a5 12861035

[B20] Winkler E. A. Bell R. D. Zlokovic B. V. (2011). Central nervous system pericytes in health and disease. Nat. Neurosci. 14, 1398–1405. 10.1038/nn.2946 22030551 PMC4020628

[B21] Wolswijk G. (1998). Chronic stage multiple sclerosis lesions contain a relatively quiescent population of oligodendrocyte precursor cells. J. Neurosci. 18, 601–609. 10.1523/JNEUROSCI.18-02-00601.1998 9425002 PMC6792542

[B22] Zawadzka M. Rivers L. E. Fancy S. P. Zhao C. Tripathi R. Jamen F.. (2010). CNS-resident glial progenitor/stem cells produce Schwann cells as well as oligodendrocytes during repair of CNS demyelination. Cell Stem Cell 6, 578–590. 10.1016/j.stem.2010.04.002 20569695 PMC3856868

