# Supplementary material for: Pericytes Favor Oligodendrocyte Fate Choice in Adult Neural Stem Cells
Source: Front Cell Neurosci. 2019 Mar 27;13:85. doi: 10.3389/fncel.2019.00085 (PMC6446960; doi:10.3389/fncel.2019.00085)
Supplement: Supplementary file 1 [file Table_1.DOCX]

**Supplemental Material and Methods**

1. ***Animals***

All experiments were conducted in accordance with the Chilean Government's Manual of Bioethics and Biosafety (CONICYT: The Chilean Commission of Scientific and Technological Research, Santiago of Chile, Chile) and according to the guidelines established by the Animal Protection Committee of the Universidad Austral de Chile (Approved Number: Informe 258/2016). Animals were handled in accordance with the guidelines of the National Institutes of Health Guide for Care and Use of Laboratory Animals and approved by the Institutional Animal Care and Use Ethics Committee of the Universidad Austral de Chile. In addition, animal handling for primary cell culture preparations was also performed in accordance with Austrian laws on animal experimentation and were approved by Austrian regulatory authorities (Permit No. BMWF-66.012/0001-II/3b/2014; license codes BMBF-66-012/0037-WF/V/3b/2014 and BMWF-66.012/0032-WF/V/3b/2015).

1. ***Preparation of primary bone marrow-derived mesenchymal stem cells***

Rat bone marrow-derived mesenchymal stem cells (MSCs) were prepared as previously described in (Rivera et al., 2006). Briefly, 6 – 8 weeks old female Fisher 344 rats were anesthetized by Isofluran and sacrificed. Bones were isolated from the hind limbs and bone marrow was washed out and dissociated in a petri dish by syringe with alphaMEM. Bone marrow was washed twice with alphaMEM and seeded in a P100 petri dish in alphaMEM containing 1% Pen/Strep and 10% FBS and cultured in a humidifying incubator (20% O_2_, 5% CO_2_ at 37°C).

1. ***Preparation of primary CNS pericytes***

Briefly, rat primary pericytes (PCs) were isolated from 6 – 8 weeks old female Fisher 344 rats. 5 Rats were anesthetized withby Isofluran and subsequently decapitated. Brains were collected in ice cold alphaMEM (Gibco). Meninges were removed properly and brains were minced in alphaMEM by with a douncer until homogenization. Half of a centrifugation tube was filled with 4°C cold 15% dextran solution in alphaMEM (autoclaved) and carefully overlayed with brain homogenate and centrifuged at 5000 g at 4°C for 10 min. Pellet containing cerebral vessels was collected and washed once with alphaMEM (1000 g, 10 min, 4°C). Vessels were resuspended in alphaMEM and filtered through a 150 µm mesh and the flow-through was again filtered through a 40 µm mesh to get rid ofeliminate single cells. Microvessels were collected in alphaMEM. After centrifugation (1000 g, 10 min, 4°C), microvessels were digested in a waterbath for 30 min at 37°C with 0.01% papain (Worthington Biochemicals, England), 0.1% dispase II (Sigma), 0.01% DNase I (Worthington Biochemicals), 0.1% collagenase 1 (Life Technologies) and 12.4 mM MgSO4 in HBSS without Mg2+/Ca2+ (Life Technologies). Every 10 minutes digested microvessels were vortexed. Digested microvessels were centrifuged (1000 g, 10 min), cells were cultured in 1 well of a 12 well plate in alphaMEM containing 20% FBS (Gibco) and 1% Penicillin/ Streptomycin (Thermo Fisher) in a humidifying incubator (20% O2, 5% CO2 at 37°C). After first passage cells were incubated in alphaMEM containing 10% FBS.

1. ***Collection of conditioned media***

PCs and MSCs were seeded each in a cell density of 12,000 cells per cm^2^ and incubated in the appropriate medium. After 3 days of incubation, medium was filtered using a 0.22 µm filter.

1. ***Immunocytochemistry***

Fixed NSCs were washed in TBS (0.15 M NaCl, 0.1 M Tris-HCl, pH 7.5), then blocked with solution composed of TBS; 0.1% Triton-X100 (only for intracellular antigens); 1% bovine serum albumin (BSA) and 0.2% Teleostean gelatin (Sigma, Germany) (fish gelatin buffer, FGB). The same solution was used during the incubations with antibodies. Primary antibodies were applied overnight at 4°C. Fluorochrome-conjugated species-specific secondary antibodies were used for immunodetection. The following antibodies and final dilutions were used. Primary antibodies: rabbit anti-GFAP 1:1000 (Dako, Denmark); mouse anti-Myelin Basic Protein (MBP) 1:750 (SMI-94, Covance, Anopoli Biomedical Systems, Eichgraben, Austria); rabbit anti-Galactocerebroside (GalC) 1:200 (Millipore) rabbit anti Ki67 1:500 (Thermos Sc.); goat anti olig2 1:200 (Abcam);. Secondary antibodies: donkey anti-mouse, rabbit, goat conjugated with Alexa Fluor 488, Alexa 568 1:1000 (Molecular Probes, Eugene, OR, USA). Nuclear counterstaining was performed with 4´, 6´-diamidino-2-phenylindole dihydrochloride hydrate at 0.25 μg/μl (DAPI; Sigma, Germany). Specimens were mounted on microscope slides using in Prolong Antifade kit (Molecular Probes, U.S.A.) or, alternatively, Dako Fluorescence Mountain Medium (Dako, Denmark). Epifluorescence observation and photo-documentation were realized using an Olympus IX81 microscope (Olypmpus, Germany) equipped with Hamamatsu digital camera and Volocity software (Perkin Elmer, Germany). Alternatively, photo-documentation was performed using an Olympus BX51 microscope (Olypmpus, Germany) or a LEICA DM 2000 Led (Leica, Germany) equipped with a digital camera and Image-pro software (Media Cybernetics, USA) and Ocular Software respectively (QImaging, Canada).

1. ***Quantitative gene expression analyses***

NSCs were seeded in 100 μg/mL poly-l-ornithine (Sigma) and 5 μg/mL Laminin (Sigma) coated petri dishes at a density of 2×10^4^ cells/cm^2^. The cells were treated with control media, MSC-CM and PC-CM respectively for 3 days *in vitro*. RNA was isolated by Tri Reagent (Sigma) according to the manufacturer’s instructions. The cDNA synthesis was performed with iScript™ Reverse Transcription Supermix for RT-qPCR (Biorad). Quantitative gene expression analyses were performed using TaqMan RT-PCR technology. Technical duplicates containing 10ng of reverse transcribed RNA were amplified with the GoTAQ Probe qPCR Master Mix (Promega) using a two-step cycling protocol (95°C for 15 sec, 60°C for 60 sec; 40 cycles, Bio-Rad CFX 96 Cycler). Following gene expression assays were employed: Id2 (Rn01495280_m1, Applied Biosystems), Olig2 (Rn0056603_m1, Applied Biosystems), DCX (Rn00584505_m1, Applied Biosystems) as well as the following validated housekeepers: Eef2 (Rn.PT.56a.36171938.gs, Integrated DNA Technologies), Psmd4 (Rn.PT.56a.18210824, Integrated DNA Technologies), Ywhaz (Rn.PT.56a.8368619, Integrated DNA Technologies). Quantification analyses were performed with qBase Plus (Biogazelle) using geNorm algorithms for multi-reference gene normalization followed by normalization to control conditions.

1. ***MTT proliferation assay***

Cell proliferation and survival was measured with 3-(4,5-Dimethyl-2-thiazolyl)-2,5-diphenyl-2H-tetrazolium bromide (MTT). NSCs were seeded onto 96 well plates at a density of 5x103 cells per well and incubated with α MEM and PCs-CM media during 0, 24, and 48 hours. The medium was replaced with 100 µl of α MEM without serum and 20 µl of MTT (5 mg/ml, M2128, sigma) and incubated during 4 hours at 37°C. After the incubation period, the medium was replaced with 100 µl of DMSO. The optical density was measured at a wavelength of 570 nm using a microplate reader (tecan infinite 200 PRO).

**References**

Rivera, F.J., Couillard-Despres, S., Pedre, X., Ploetz, S., Caioni, M., Lois, C., et al. (2006). Mesenchymal Stem Cells Instruct Oligodendrogenic Fate Decision on Adult Neural Stem Cells. *STEM CELLS* 24(10)**,** 2209-2219.
